# Comparison of the effects of zoledronic acid versus denosumab on bone metabolism, inflammatory response, and immunoglobulins in breast cancer patients with bone metastases

**DOI:** 10.5937/jomb0-56984

**Published:** 2025-07-04

**Authors:** Xian Zhang, Jinfeng Tong, Zhi Wang, Hailong Yang, Pei Shi

**Affiliations:** 1 Jiujiang NO.1 People's Hospital, Jiujiang City Key Laboratory of Cell Therapy, Department of Oncology, Jiujiang, Jiangxi, China; 2 Jiujiang NO.1 People's Hospital, Jiujiang City Key Laboratory of Cell Therapy, Department of Clinical Laboratory, Jiujiang, Jiangxi, China; 3 Jiujiang NO.1 People's Hospital, Jiujiang City Key Laboratory of Cell Therapy, Department of Orthopedics, Jiujiang, Jiangxi, China; 4 First Affiliated Hospital, Heilongjiang University of Chinese Medicine, Department of Oncology, Haerbin, Heilongjiang, China

**Keywords:** zoledronic acid, denosumab, bone metastases, breast cancer, bone metabolism, zoledronska kiselina, denosumab, metastaze u kostima, rak dojke, metabolizam kostiju

## Abstract

**Background:**

This study aimed to compare the effects of Zoledronic Acid (ZA) versus Denosumab (DEN) on bone metabolism, inflammatory response, and immunoglobulins in breast cancer (BC) patients with bone metastases.

**Methods:**

The potential study population consisted of 163 patients with bone metastases BC admitted from May 2023 to June 2024. Through propensity score matching (PSM), 122 patients were included, with 61 patients in the ZA group (treated with ZA) and 61 patients in the DEN group (treated with DEN). Levels of tartrate-resistant acid phosphatase 5b (TRACP-5b), bone glutamyl protein (BGP), bone alkaline phosphatase (BALP), and 25-hydroxyvitamin D3 (25(OH)D3) were measured before and after treatment. Additionally, bone mineral density (BMD) of the lumbar spine (L1-L4) and hip, as well as levels of inflammatory factors and immunoglobulins, were assessed.

**Results:**

After PSM, baseline characteristics were balanced between the ZA and DEN groups (P > 0.05). No significant difference was observed in post-treatment BMD between the two groups (P > 0.05). However, the DEN group showed significantly lower TRACP-5b and BGP levels and notably higher BALP and 25(OH)D3 levels compared to the ZA group (P <0.05). In contrast, the ZA group exhibited lower levels of inflammatory factors and higher immunoglobulin levels than the DEN group (P < 0.05). Regarding safety, a lower incidence of adverse reactions was determined in the DEN group versus the ZA group (P < 0.05).

**Conclusions:**

In the treatment of BC with bone metastases, DEN demonstrated superior benefits for bone metabolism, while ZA showed better regulation of inflammatory response and immune function.

## Abstract (sr)

**Uvod:**

Ova studija je imala za cilj da uporedi efekte zoledronske kiseline (ZA) u odnosu na denosumab (DEN) na metabolizam kostiju, inflamatorni odgovor i imunoglobuline kod pacijenata sa karcinomom dojke (BC) sa metastazama u kostima.

**Metode:**

Potencijalnu studijsku populaciju činilo je 163 pacijenta sa metastazama u kostima BC primljenih od maja 2023. do juna 2024. Kroz podudaranje sklonosti (PSM), uključeno je 122 pacijenta, sa 61 pacijentom u ZA grupi (lečeni ZA) i 61 pacijentom u DEN grupi (lečeni DEN). Nivoi kisele fosfataze 5b (TRACP-5b), glutamil proteina kostiju (BGP), alkalne fosfataze kostiju (BALP) i 25-hidroksivitamina D3 (25(OH)D3) mereni su pre i posle tretmana. Pored toga, procenjena je mineralna gustina kostiju (BMD) lumbalne kičme (L1-L4) i kuka, kao i nivoi inflama-tornih faktora i imunoglobulina.

## Introduction

Breast cancer (BC) has emerged as one of the most prevalent malignancies globally. According to the Global Cancer Burden data, approximately 2.26 million new cases of BC were reported in 2020 [1]. BC is characterized by its highly aggressive and invasive nature, predisposing it to metastasis [2]. Among the common metastatic sites, the skeletal system is frequently affected, with studies showing that 55–75% of advanced BC patients develop bone metastases [3]. BC bone metastases are predominantly osteolytic, often resulting in skeletal-related events (SREs) such as pathological fractures and spinal cord compression. These complications not only disrupt treatment regimens but also significantly increase the risk of adverse outcomes [4]. To mitigate these risks and improve patient prognosis, clinical guidelines strongly recommend the prompt use of bone-modifying agents following the diagnosis of bone metastases [5].

Zoledronic Acid (ZA), a representative bisphosphonate, is one of the commonly used drugs for bone metastasis. However, its clinical application is limited by a relatively short duration of therapeutic efficacy and a higher incidence of adverse effects such as fever and renal impairment [6]. In contrast, Denosumab (DEN), a novel bone-modifying agent, is a human immunoglobulin (Ig) G2 monoclonal antibody that specifically targets receptor activator of nuclear factor-kB ligand (RANKL) with high affinity. This unique mechanism of action enables DEN to effectively inhibit osteoclast differentiation and activation, consequently suppressing bone resorption [7]. Comparative studies in osteoporosis treatment have demonstrated comparable efficacy between ZA and DEN, with DEN potentially offering additional cardiovascular benefits [8]. Moreover, in multiple myeloma patients, DEN has shown superior efficacy in reducing skeletal-related events (SREs) [9]. These findings suggest that DEN may represent a more advantageous treatment option for patients with tumor-derived bone metastases. However, for BC patients with bone metastases, the clinical evidence remains limited, with only one relevant clinical trial conducted by Stopeck et al. [10] in 2010 providing reference data.

Given this background, the present study aims to systematically compare the effects of ZA and DEN on bone metabolism, inflammatory responses, and Igs in BC patients with bone metastases. Through elucidating the mechanistic differences and therapeutic outcomes of these two agents, we endeavor to provide clinicians with robust scientific evidence for optimizing individualized treatment strategies, ultimately enhancing therapeutic outcomes and quality of life for BC patients with bone metastases.

## Materials and methods

### Study design

This study was designed as a retrospective analysis, utilizing propensity score matching (PSM) to improve the reliability of the findings. First, we calculated the sample size needed for the study (effect size=0.5, α=0.05, power=0.95) by using G-Power (v3.1) software, and the output showed that a minimum of 42 study subjects were needed for each group. The study population included BC patients with bone metastases who were admitted to our hospital between May 2023 and June 2024.

Inclusion criteria:

- Patients aged 18 years or older;
- Histologically confirmed diagnosis of BC;
- Radiographic evidence of at least one bone metastasis;
- Normal results on routine blood tests and liver/kidney function tests;
- No contraindications to the proposed treatments;
- Availability of complete medical records;
- An expected survival of at least 6 months.

Exclusion criteria:

- Presence of metabolic bone diseases or vitamin deficiencies;
- Comorbid conditions such as drug metabolism disorders, other malignancies, or chronic liver/kidney diseases;
- Secondary osteoporosis;
- Known allergies to the medications used in this study;
- Prior use of medications known to affect bone metabolism.
- Patients who died or were lost to visit during follow-up.

### Data collection

Following screening according to the inclusion and exclusion criteria, 163 patients were initially enrolled. Detailed demographic and clinical data were collected, including age, menopausal status, tumor type, sites of metastasis, and antitumor treatment regimens. PSM was performed to balance baseline characteristics between groups, with a standardized mean difference (SMD) threshold of < 0.1 [11]. The study involving human subjects complied with the Declaration of Helsinki and was approved by the ethical committee of the Jiujiang First People’s Hospital (No. JJSDYRMYY-YXLL-2021-086), and all participants provided written informed consent [12].

### Treatment protocol

For hormone receptor-positive (ER+/PR+) BC patients, endocrine therapies such as Tamoxifen and Letrozole were given; for HER2-positive BC patients, targeted therapies such as Trastuzumab and Lapatinib were given; and for hormone receptor-negative BC patients with rapid disease progression or severe symptoms, the Capecitabine, Gemcitabine and other chemotherapy. Calcium (1200 mg/day) and vitamin D (800-1000 IU/day) supplementation.

- ZA (Shenzhen Neptunus Pharmaceutical Co., Ltd., H20041957): ZA was administered intravenously at a dose of 4 mg per session, diluted in 100 mL of normal saline. The infusion was delivered over a minimum of 15 minutes, with treatments repeated every 28 days for a total of 6 cycles.

- DEN (Jiangsu Taikang Biopharmaceutical Co., Ltd., S20233111): DEN was administered subcutaneously at a dose of 120 mg per session, repeated every 28 days for 6 cycles.

### Laboratory tests

Fasting venous blood samples were collected from patients before and after treatment. Serum was isolated by centrifugation and divided into three aliquots for analysis: 1. The first aliquot was analyzed using an automated biochemical analyzer (BS-350E, Myriad) to measure levels of hypersensitive C-reactive protein (hs-CRP) and 25-hydroxyvitamin D_3_ (25(OH)D_3_). 2. The second aliquot was assessed using enzyme-linked immunosorbent assay (ELISA) to quantify levels of tartrate-resistant acid phosphatase 5b (TRACP-5b), bone glutamyl protein (BGP), bone alkaline phosphatase (BALP), tumor necrosis factor-α (TNF-α), interleukin-1β, -6, and -8 (IL-1β, IL-6, IL-8), and interferon-γ (IFN-γ) (The kits were purchased from Wuhan Elabscience Bio-technology Co.), the operation process was carried out in strict accordance with the kit instructions. 3. The third aliquot was analyzed using a chemiluminescence immunoassay (E411, Roche) to determine levels of Igs (IgA, IgG, IgE, and IgM).

Bone mineral density (BMD) of the lumbar spine (L1-L4) and hip was measured using dual-energy X-ray absorptiometry (DXA) (SOMATOM Force, Siemens) before treatment initiation and after 6 months of treatment.

### Statistical snalysis

Data were analyzed using SPSS 27.0 and R language 4.3.1. PSM was employed to match patients treated with either ZA or DEN in a 1:1 ratio. Continuous variables were expressed as mean ± standard deviation (x̄±s), with between-group comparisons performed using independent t-tests and withingroup comparisons assessed using paired t-tests. Categorical variables were expressed as percentages (%), and between-group comparisons were analyzed using chi-square tests. A P-value < 0.05 was considered statistically significant.

## Results

### Baseline characteristics of patients and PSM

Of the 163 patients included in the study, 89 were treated with ZA (ZA group) and 74 with DEN(DEN group). Prior to PSM, baseline characteristics between the two groups showed a statistically significant difference in menopausal status (*P* < 0.05). Among all variables, only visceral metastasis and the use of combined chemotherapy had an SMD < 0.1. After PSM, 122 patients were matched, with 61 cases in each of the ZA and DEN groups. Post-PSM analysis demonstrated no significant differences in baseline characteristics between the two groups (*P* > 0.05), with all SMD values < 0.1 (Table 1).

**Table 1:** Baseline Characteristics of Patients and PSM.

Projects	Before PSM			After PSM		
	ZA group<br>(n=89)	DEN group<br>(n=74)	t (or χ^2^)/<br>SMD/P	ZA group<br>(n=61)	DEN group<br>(n=61)	t (or χ^2^)/<br>SMD/P
Age	57.11±9.10	55.19±10.82	1.233/0.121/<br>0.220	54.87±7.10	53.11±9.86	1.128/0.827/<br>0.262
Duration of disease<br>(months)	3.56±0.90	3.69±0.98	0.863/0.147/<br>	3.54±0.89	3.72±0.97	1.073/0.496/<br>0.286
Menstrual status			4.026/0.315/<br>0.045			0.038/0.035/<br>0.846
menopausal	65 (73.03)	45 (58.11)		42 (68.85)	41 (67.21)	
non-menopausal	24 (26.97)	31 (41.89)		19 (31.15)	20 (32.79)	
Visceral metastasis			0.920/0.151/<br>0.338			0.132/0.066/<br>0.717
yes	50 (56.18)	36 (48.65)		30 (49.18)	28 (45.90)	
no	39 (43.82)	38 (51.35)		31 (50.82)	33 (54.10)	
Combined targeted<br>therapy			1.354/-0.183<br>/0.245			0.153/-0.071/<br>0.696
yes	51 (57.30)	49 (66.22)		41 (67.21)	43 (70.49)	
no	38 (42.70)	25 (33.78)		20 (32.79)	18 (29.51)	
Combined endocrine<br>therapy			0.597/0.122/<br>0.440			0.145/0.069/<br>0.703
yes	39 (43.82)	28 (37.84)		22 (36.07)	20 (32.79)	
no	50 (56.18)	46 (62.16)		39 (63.93)	41 (67.21)	
Combined<br>chemotherapy			0.256/0.080/<br>0.613			0.034/0.033/<br>0.854
yes	54 (60.67)	42 (56.76)		36 (59.02)	35 (57.38)	
no	35 (39.33)	32 (43.24)		25 (40.98)	26 (42.62)	
Number of bone<br>metastases			2.190/0.243/<br>0.139			0.185/0.073/<br>0.667
1	61 (62.24)	54 (72.97)		48 (78.69)	46 (75.41)	
2	37 (37.76)	20 (27.03)		13 (21.31)	15 (24.59)	
Site of bone metastasis			1.460/0.153/<br>0.227			0.137/0.070/<br>0.711
lumbar vertebra	56 (57.14)	49 (66.22)		36 (59.02)	38 (62.30)	
thoracic vertebra	42 (42.86)	25 (33.78)		25 (40.98)	23 (37.70)	

### Comparison of bone metabolism and BMD

Tumor-derived bone metastasis disrupts the normal equilibrium of bone metabolism, giving rise to augmented bone resorption and diminished bone formation. Enhanced osteoclast activity and suppressed osteoblast function result in bone destruction, triggering SREs [12]. Therefore, bone metabolism markers and BMD before and after treatment served as the primary outcome measures in this study. At baseline, no significant differences were observed in bone metabolism markers or BMD between the two groups (*P* > 0.05). Following treatment, the DEN group demonstrated significantly lower levels of TRACP-5b and BGP, along with higher levels of BALP and 25(OH)D_3_, compared to the ZA group (*P* < 0.05). When compared to pretreatment values, both groups exhibited a reduction in TRACP-5b and BGP, as well as an increase in BALP and 25(OH)D_3_ after treatment (*P* < 0.05). Regarding BMD, both groups showed an increase in lumbar spine (L1-L4) and hip BMD post-treatment compared to baseline (*P* > 0.05). However, no significant inter-group differences in BMD were observed after treatment (*P* > 0.05) (Table 2).

**Table 2:** Comparison of Bone Metabolism and BMD. Note: comparison with before treatment #P<0.05.

Projects		Group ZA (n=61)	Group DEN (n=61)	t	P
TRACP-5b (U/L)	Before treatment	5.61±1.09	5.73±1.51	0.497	0.062
	After treatment	3.85±1.14#	3.46±0.72#	2.215	0.029
BALP (U/L)	Before treatment	99.49±12.14	101.79±15.15	0.925	0.357
	After treatment	108.09±12.81#	113.29±14.53#	2.095	0.038
BGP (ng/mL)	Before treatment	15.65±2.82	15.96±2.79	0.610	0.543
	After treatment	13.67±2.82#	10.87±2.31#	5.985	<0.001
25(OH)D_3_ (ng/mL)	Before treatment	20.87±5.16	19.48±4.01	1.666	0.098
	After treatment	23.98±4.70#	26.44±4.99#	2.809	0.006
BMD of the lumbar<br>spine (L1-L4) (g/cm^2^)	Before treatment	0.78±0.20	0.76±0.16	0.753	0.453
	After treatment	0.93±0.14#	0.89±0.14#	1.683	0.095
BMD of the hip<br>(g/cm^2^)	Before treatment	0.60±0.04	0.61±0.05	1.229	0.221
	After treatment	0.78±0.06#	0.79±0.07#	0.824	0.412

### Comparison of inflammatory response

Abnormal bone metabolism can initiate a systemic inflammatory response and impact immune function [13]. Thus, we compared the inflammatory responses of the two patient groups. Results indicated that prior to treatment, no differences were found in hs-CRP, IL-1β, IL-6, IL-8, TNF-α, and IFN-γ between the groups (*P* > 0.05). However, post-treatment, the ZA group had lower levels of hs-CRP, IL-1β, IL-6, IL-8, and TNF-α compared to the DEN group (*P* < 0.05) (Table 3).

**Table 3:** Comparison of Inflammatory Response. Note: comparison with before treatment #P<0.05.

Projects		Group ZA (n=61)	Group DEN (n=61)	*t*	*P*
hs-CRP (mg/L)	Before treatment	28.75±4.51	29.72±5.46	1.070	0.287
	After treatment	24.35±5.33#	27.39±6.00#	3.250	0.002
IL-1β (pg/mL)	Before treatment	45.56±6.10	46.98±7.53	1.150	0.252
	After treatment	37.67±5.52#	40.87±6.37#	2.968	0.004
IL-6 (pg/mL)	Before treatment	34.21±6.62	36.21±6.57	1.674	0.097
	After treatment	28.58±4.56#	31.90±5.39#	3.672	<0.001
IL-8 (pg/mL)	Before treatment	23.06±4.17	23.00±3.60	0.083	0.934
	After treatment	18.45±3.54#	20.73±3.81#	3.427	<0.001
TNF-α (pg/mL)	Before treatment	67.74±6.36	68.20±5.93	0.412	0.681
	After treatment	61.27±5.61#	63.45±5.96#	2.076	0.040

### Comparison of Igs

Similarly, no pre-treatment differences in Igs were observed between the two groups (*P* > 0.05). After treatment, levels of IgA, IgG, IgM, and IgE increased in both groups, with the ZA group showing higher levels than the DEN group (*P* < 0.05) (Table 4).

**Table 4:** Comparison of Igs. Note: comparison with before treatment #P<0.05.

Projects		Group ZA (n=61)	Group DEN (n=61)	*t*	*P*
IgA (g/L)	Before treatment	0.55±0.10	0.58±0.09	1.627	0.106
	After treatment	0.72±0.10	0.68±0.09	2.332	0.021
IgG (g/L)	Before treatment	6.43±0.84	6.67±0.93	1.524	0.130
	After treatment	7.85±1.49	7.06±1.23	3.193	0.0.02
IgM (g/L)	Before treatment	0.53±0.09	0.55±0.08	1.103	0.272
	After treatment	0.74±0.13	0.64±0.08	5.400	<0.001
IgE (mg/L)	Before treatment	0.35±0.09	0.33±0.06	0.904	0.368
	After treatment	0.46±0.12	0.40±0.08	3.022	0.003

### Comparison of safety

Regarding safety, no statistical differences were noted in the incidence of hypocalcemia, renal function impairment, or muscle soreness between the two groups (*P *> 0.05). Nevertheless, the DEN group had a lower incidence of joint pain and fever than the ZA group (*P* < 0.05) (Table 5).

**Table 5:** Comparison of Safety.

Projects	Group ZA (n=61)	Group DEN (n=61)	*χ^2^*	*P*
Fever	11 (11.48)	1 (1.64)	4.816	0.028
Nausea and vomiting	3 (4.92)	2 (3.28)	0.209	0.648
Kidney impairment	0 (0.00)	1 (1.64)	1.008	0.315
Muscle aches and pains	3 (4.92)	3 (4.92)	-	-
Joint pain	9 (14.75)	2 (3.28)	4.896	0.027
Hypocalcemia	8 (13.11)	7 (11.48)	0.076	0.783
Osteonecrosis of the jaw	1 (1.64)	0 (0.00)	1.008	0.315

## Discussion

With the rising global incidence of BC, bone metastases have emerged as a prevalent clinical complication, significantly impacting patient management and outcomes [14]. Our results suggest that DEN can better improve bone health in patients with BC bone metastases, while ZA can better inhibit the inflammatory response in patients with BC bone metastases, and these results provide new references for the treatment of BC bone metastases in the future.

This comparative study evaluated the therapeutic efficacy of ZA and DEN in BC patients with bone metastases. It is important to note that as a retrospective analysis, our study was influenced by specific contextual factors in the Chinese healthcare setting. DEN received clinical approval in China in May 2019, and its utilization has been constrained by higher costs and limited accessibility compared to the more established ZA. To address potential confounding variables and enhance the validity of our findings, we implemented PSM between BC patients with bone metastases treated with either DEN or ZA, thereby improving the comparability of our study groups. After PSM, the baseline characteristics of the two groups were well-balanced, with all SMD values below 0.1, which ensured comparability. In the subsequent comparison of bone metabolism and BMD, although pre- and post-treatment BMD didn’t differ significantly between the ZA and DEN groups, the DEN group exhibited lower levels of TRACP-5b and BGP, and higher levels of BALP and 25(OH)D_3_. These results suggest that DEN may be more effective in improving bone metabolism in patients. TRACP-5b is a reliable bone resorption marker, reflecting bone resorption and osteoclast activity [15]. BALP, a specific product of bone formation, can reliably indicate osteoblast activity [16]. BGP is secreted by osteoblasts and chondrocytes, contributing to increased bone mineral content and reflecting osteoblast activity [17]. 25(OH)D_3_, converted from vitamin D3 by 25-hydroxylase,reflects the body’s vitamin D storage. Higher levels of vitamin D storage are conducive to enhancing osteoblast activity and promoting bone formation [18]. Based on these findings, we conclude that DEN has a stronger anti-bone resorption effect than ZA, which can reach the bone surface via the circulatory system and remodel trabecular bone. In contrast, ZA, as a representative bisphosphonate, has a relatively weak inhibitory effect on osteoclasts, which may explain DEN’s more significant improvement in patients’ bone metabolism. However, ZA, as a classic bone resorption inhibitor, has been repeatedly validated for its positive impact on skeletal health [19] [20] [21]. Although ZA’s effect on bone metabolism is less significant than that of DEN, it may still help maintain BMD to the greatest extent possible. However, the improvement in bone metabolism did not translate into an elevated BMD, and we, on the other hand, believe that the reasons for this may be as follows: (1) The bone remodeling cycle is long, and the dynamic balance of bone metabolism (bone formation and bone resorption) usually takes months or even years to be reflected in BMD [22], whereas the interval in the present study was only 6 months. (2) Secondly, bone metabolism may be active in specific areas (e.g. repair of microfractures) without significant changes in overall BMD. (3) If bone formation markers are elevated but bone resorption markers are increased in parallel, this may create a bone formation and resorption balance, resulting in an elevated bone conversion rate but unchanged net bone mass [23]. (4) Finally, dual-energy X-ray absorptiometry (DXA) is less sensitive to small BMD changes (<3–5%) and may not capture early improvements, and quantitative CT (QCT) or high-resolution peripheral CT (HR-pQCT) may be more sensitive to assess bone microarchitecture [24]. Therefore, more accurate results may be obtained using more sophisticated instruments.

Subsequently, we delved deeper into differences in inflammatory responses and Igs between the two patient groups. The results revealed that post-treatment, the ZA group had lower levels of inflammatory factors and higher levels of Igs than the DEN group. Immunoglobulin is an important component of the immune system, inducing tumor cell killing by binding to antigens that can bind to the surface of tumor cells, and to natural killer cells (NK cells) or macrophages through the Fc segment [25]. Therefore, elevated immunoglobulin levels largely predict that the patient’s in vivo anti-tumor status becomes better. This suggests that ZA is more effective in ameliorating the inflammatory response and immune function of patients. The reasons for these findings can be analyzed as follows: ZA not only directly inhibits the release of pro-inflammatory cytokines, thereby reducing inflammation, but also mitigates inflammation by decreasing bone destruction and the associated inflammatory response [26]. Conversely, DEN has a relatively feeble direct impact on inflammatory factors and primarily alleviates inflammation indirectly through reducing bone destruction [27]. In an in-vitro study by Lo Presti E et al. [28], ZA was found to enhance antitumor immune responses by activating γδ T cells. DEN, on the other hand, mainly influences the immune response by blocking the regulatory effect of RANKL on immune cells [29]. Therefore, compared with DEN, ZA demonstrates more prominent anti-inflammatory and immunomodulatory effects. However, in a study on periodontitis mice, Kuritani M et al. reported that DEN showed a more pronounced anti-inflammatory effect than ZA [30], which contradicts the findings of our current study. We postulate that this discrepancy may be attributed to two factors. Firstly, the nature of the diseases differs. Secondly, radiotherapy and chemotherapy in BC patients with bone metastases can exacerbate inflammatory res ponses and induce more pronounced immunosuppression, which differs significantly from the underlying pathological manifestations of periodontitis. Consequently, the effects of ZA and DEN may also vary markedly. Nonetheless, this hypothesis necessitates prompt in vitro experiments to clarify the underlying mechanisms of the two drugs.

Finally, in the safety comparison, we found that DEN had milder drug side effects than ZA, which isconsistent with our expected results. Similarly, many previous comparative studies on ZA and DEN have consistently demonstrated the higher therapeutic safety of DEN [31] [32] [33]. Additionally, since ZA inhibits the mevalonate pathway, it may potentially have an indirect impact on tumor cell immune evasion [34]. Therefore, in clinical practice, personalized decision-making remains essential, taking into account the specific conditions of each patient.

As this study employed a retrospective design, inherent selection bias resulted in significant imbalances in baseline characteristics between the two patient cohorts. While PSM was implemented to enhance the comparability of baseline characteristics between groups, the fundamental limitations associated with retrospective analyses precluded comprehensive adjustment for all potential confounding factors. Consequently, these findings necessitate validation through rigorously designed randomized controlled trials to establish their reliability. Furthermore, the relatively short study period limited our ability to observe long-term adverse events and patient adherence to ZA and DEN. Also, since this study was conducted in patients with BC bone metastases, antitumor therapy for patients was unavoidable. And whether these treatments will interfere with the effect of ZA or DEN is still unknown. In subsequent studies, we should confirm the effects of ZA and DEN on bone status by in vitro tests. These limitations are issues that need to be addressed in our subsequent research.

## Conclusion

Compared to ZA, DEN is more beneficial for the bone metabolic health of BC patients with bone metastases. However, ZA demonstrates superior efficacy in regulating inflammatory responses and immune function in patients. Therefore, when treating BC bone metastases in the future, clinical recommendations for ZA are preferred for patients with a severe inflammatory state, while DEN is recommended for patients with severe bone resorption, and these decisions are expected to further improve the prognostic health of patients.

## Dodatak

### Ethical approval

The study involving human subjects complied with the Declaration of Helsinki and was approved by the ethical committee of the Jiujiang First People’s Hospital (No.JJSDYRMYY-YXLL-2021-086), and all participants provided written informed consent.

### Data availability statement

The data used to support the findings of this study are available from the corresponding author upon request.

### Funding statement

The authors did not receive specific funding.

### Consent to publish

All authors gave final approval of the version to be published.

### Conflict of interest statement

All the authors declare that they have no conflict of interest in this work.
